# Defects in Thymocyte Differentiation and Thymocyte- Stromal Interactions in the Trisomy 16 Mouse

**DOI:** 10.1155/1992/72627

**Published:** 1992

**Authors:** Janet L. Ewart, Robert Auerbach

**Affiliations:** 1 Department of Biology Goddard Laboratories University of Pennsylvania Philadelphia Pennsylvania 19104 USA; 2 Center for Developmental Biology University of Wisconsin 1117 W. Johnson Street Madison Wisconsin 53706 USA

**Keywords:** Trisomy 16, Down Syndrome, T-cell development, differentiation antigens, flow cytometry

## Abstract

We have examined fetal thymic development in the trisomy 16 (Ts16) mouse, which is considered to be a model for human trisomy 21, or Down Syndrome. The Ts16 thymus contains 10 to 20% of the number of lymphocytes found in a normal thymus at a comparable stage. Expression of thymocyte differentiation markers (Thy-1, CD5, CD8, CD4, CD3, and HSA) is severely affected in Ts16 fetuses aged 14–18 gestational days. When thymuses from 14-day Ts16 mice were cultured *in vitro*, these markers eventually reached levels of expression comparable to those seen in normal thymuses in culture. On the other hand, expression of CD44 appears to be unaffected in Ts16 thymuses *in vivo*, but declines *in vitro* relative to normal thymuses. Reconstitution of depleted thymic stroma with thymocytes showed evidence of defects in both developmental compartments.


*Developmental Immunology*, 1992, Vol. 2, pp. 215–226
Reprints available directly from the publisher
Photocopying permitted by license only

© 1992 Harwood Academic Publishers GmbH
Printed in the United Kingdom

### Defects in Thymocyte Differentiation and Thymocyte-Stromal Interactions in the Trisomy 16 Mouse

JANET L. EWART† and ROBERT AUERBACH*‡

†*Department of Biology, Goddard Laboratories, University of Pennsylvania, Philadelphia, Pennsylvania 19104*
‡*Center for Developmental Biology, University of Wisconsin, 1117 W. Johnson Street, Madison, Wisconsin 53706*

We have examined fetal thymic development in the trisomy 16 (Ts16) mouse, which is considered to be a model for human trisomy 21, or Down Syndrome. The Ts16 thymus contains 10 to 20% of the number of lymphocytes found in a normal thymus at a comparable stage. Expression of thymocyte differentiation markers (Thy-1, CD5, CD8, CD4, CD3, and HSA) is severely affected in Ts16 fetuses aged 14–18 gestational days. When thymuses from 14-day Ts16 mice were cultured *in vitro*, these markers eventually reached levels of expression comparable to those seen in normal thymuses in culture. On the other hand, expression of CD44 appears to be unaffected in Ts16 thymuses *in vivo*, but declines *in vitro* relative to normal thymuses. Reconstitution of depleted thymic stroma with thymocytes showed evidence of defects in both developmental compartments.

KEYWORDS: Trisomy 16, Down Syndrome, T-cell development, differentiation antigens, flow cytometry.

### INTRODUCTION

Human trisomy 21, or Down Syndrome (DS), is known to affect the immune system, causing high rates of leukemia incidence (Fong and Brodeur, 1987) and increased susceptibility to infectious diseases (Fryers, 1986). A variety of cellular defects has been reported in the DS immune system, including altered circulating antibody levels (Sassaman, 1982), decreased natural cytotoxic activity (Montagna et al., 1988; Nair and Schwartz, 1989), impaired T-cell responses (Montagna et al., 1988; Raziuddin and Elawad, 1990), and altered expression of differentiation antigens (Larocca et al., 1988; Noble and Warren, 1988; Raziuddin and Elawad, 1990). However, the developmental origin of these cellular alterations is not known.

Murine trisomy 16 (Ts16) has been proposed as an animal model for DS. The use of this particular trisomy is based on the fact that several genes from the region of human chromosome 21 most important in creating the DS phenotype (Sinet et al., 1976; Summitt, 1981; Antonarakis et al., 1989; Rahmani et al., 1989) are shared between human chromosome 21 and mouse chromosome 16 (Epstein et al., 1984; Reeves et al., 1986). These mice can be systematically produced by using a breeding scheme involving Robertsonian chromosome translocations (Gropp et al., 1975). Mice are produced that are heterozygous for two different Robertsonian chromosomes, each of which contains the chromosome of interest. When these animals are bred with chromosomally normal mice, their offspring may be normal, monosomic, trisomic, or combined monosomic/trisomic. The monosomics and monosomic/trisomics die by day 8 of gestation (Gropp, 1982; Epstein, 1988), leaving only trisomic and normal mice. Ts16 mice can survive until birth, but die within a few hours (Miyabara et al., 1982).

The first observation to indicate that Ts16 affects the immune system was that the thymus is only about 20% of normal size throughout development (Epstein et al., 1985). Herbst et al. (1982) examined the ability of fetal liver cells from mice carrying various trisomies to repopulate lethally irradiated animals. They found that, although some trisomies had no apparent effect on this stem-cell population, Ts16 caused significant reductions in repopulation. This suggests impaired development of Ts16 lymphocytes even before they reach the thymus or other adult

*Corresponding author.

lymphoid organs. Cox et al. (1984) fused Ts16 and normal embryos at the 8–12 cell stage. In nearly all organs of the resultant chimeras, trisomic and normal cells were present in approximately equal numbers as measured by glucose phosphate isomerase isozyme analysis. In the thymus and placenta, however, Ts16 cells were markedly deficient as compared to control chimeras made with normal littermates or to other tissues of the same animal. This is further evidence for a primary defect in cells of the immune system in Ts16 mice. Studies looking directly for defects in Ts16 lymphoid stem cells (Epstein et al., 1985) have shown reductions in stem cell numbers as measured in various *in vitro* and *in vivo* assays. However, these differences were primarily observed on a per animal basis and could be attributable to the overall smaller size of the trisomic fetus. More recently, a flow cytometric study (Berger and Epstein, 1989) has indicated developmental retardation in the Ts16 thymus. Decreased expression of T-cell markers on Ts16 thymocytes was observed, and extended organ culture of trisomic thymic rudiments resulted in the production of cells that were closer to normal thymocytes in their phenotypes.

This study was carried out in order to examine the effects of Ts16 on some of the characteristic events of T-cell development. The expression of many of the T-cell antigens was examined by flow cytometry, including multiparameter analysis to look for effects on particular subsets of cells. The Ts16 thymus was also grown in organ culture to allow study of the thymocytes after a longer period of development. Finally, thymus reconstitution was used in an effort to determine whether the observed thymic defects were due to defects in the lymphocytes or the thymic stroma.

### RESULTS

### Difference in Growth of Normal and Ts16 Thymuses

Consistent with previous reports (Epstein et al., 1985), the Ts16 thymus contains fewer thymocytes than normal throughout development (Table 1). There may be a small difference in the rate of thymic growth, but a greater part of the difference in size could be attributed to a deficit in cell number from the beginning of thymus development. This can be seen most clearly by examining the proportion of the cell numbers seen in normal versus trisomic thymuses throughout gestation (Table 1, last column). This indicates that the Ts16 thymus is initially colonized by fewer cells, with growth after this nearly paralleling that seen in normal animals. Thymuses removed at 14 gestational days (gd) and cultured *in vitro* also show a deficiency in the number of lymphocytes. Growth in culture is also parallel to that of normal thymuses, at least through 6 days *in vitro*. At 9 days, while most of the Ts16 cultures continue to increase in cell number, some (about 25%) have begun to decline in size. (This accounts for the high variability in cell number seen in these cultures.) Virtually no live cells can be recovered from Ts16 thymuses cultured for 12 days or longer.

### Expression of Thymocyte Differentiation Markers

In normal mice, expression of Thy-1 (Table 2)

TABLE 1
Number of Lymphocytes Obtained from Normal and Ts16 Thymuses

| Age[a] and genotype | | $n$ | Mean cell no. | S.D.[b] | $p$ | Proportion Ts16/normal |
|---|---|---|---|---|---|---|
| *In vivo* | | | | | | |
| 13 | Nor. | 32 | $1.0\times10^4$ | $6.7\times10^3$ | | |
| | Tri. | 26 | $2.3\times10^3$ | $1.6\times10^3$ | <0.01 | 0.23 |
| 14 | Nor. | 32 | $4.9\times10^4$ | $4.0\times10^4$ | | |
| | Tri. | 27 | $1.2\times10^4$ | $1.4\times10^4$ | <0.01 | 0.24 |
| 15 | Nor. | 36 | $2.5\times10^5$ | $1.4\times10^5$ | | |
| | Tri. | 35 | $4.6\times10^4$ | $4.5\times10^4$ | <0.01 | 0.18 |
| 16 | Nor. | 23 | $9.4\times10^5$ | $3.6\times10^4$ | | |
| | Tri. | 26 | $1.1\times10^5$ | $7.8\times10^4$ | <0.01 | 0.11 |
| 17 | Nor. | 18 | $3.8\times10^6$ | $3.2\times10^6$ | | |
| | Tri. | 15 | $6.4\times10^5$ | $1.4\times10^6$ | <0.01 | 0.19 |
| 18 | Nor. | 15 | $9.5\times10^6$ | $4.9\times10^6$ | | |
| | Tri. | 14 | $8.8\times10^5$ | $5.3\times10^5$ | <0.01 | 0.09 |
| *In vitro* | | | | | | |
| 3 | Nor. | 17 | $2.1\times10^5$ | $1.2\times10^5$ | | |
| | Tri. | 18 | $3.8\times10^4$ | $2.4\times10^4$ | <0.01 | 0.19 |
| 6 | Nor. | 12 | $6.3\times10^5$ | $2.0\times10^5$ | | |
| | Tri. | 12 | $2.0\times10^5$ | $1.5\times10^5$ | <0.01 | 0.32 |
| 9 | Nor. | 18 | $6.5\times10^5$ | $3.7\times10^5$ | | |
| | Tri. | 16 | $1.5\times10^5$ | $1.5\times10^5$ | <0.01 | 0.23 |

[a]Age indicated for cultured thymuses is the number of days in culture.
[b]S.D.=standard deviation.

rises from 13 to 16 gd, after which close to 100% of the cells express this marker through the remainder of fetal development. In the trisomic animals, a similar pattern is seen, but the proportion of cells expressing Thy-1 is lower. A small population of thymocytes (about 5%) remains Thy-1$^-$ even at 18 gd. Different results are seen *in vitro*, where essentially 100% of both normal and trisomic thymocytes label with Thy-1 after 3 or more days of organ culture.

Entry of Ts16 thymocytes into the more mature cell populations identified by expression of CD5, CD8, and CD4 appears to be delayed when compared to the course of normal development. This is particularly clear in the case of CD5 (Table 2). Expression of this antigen is close to normal levels early in development, but the increase of expression seen in normal thymuses beginning at 16 gd is delayed by a day in the Ts16 thymus and the proportion of cells expressing the antigen remains lower than normal throughout development. CD5 expression does catch up to normal after 9 days in organ culture. *In vivo*, double labeling with CD8 and CD4 shows a similar developmental retardation (Table 3; Fig. 1). The double-negative cell population remains much larger than in normal thymuses throughout development, and still is markedly enlarged at 18 days. The inverse pattern is seen in the double-positive subset, which does not show any substantial increase until the end of development. These subsets do approach normal levels after 9 days of organ culture. A different pattern, however, is seen for CD8$^+$/CD4$^-$ cells. In normal mice, cells of this phenotype (presumably representing the immature CD8-positive subset) appear at gd 16 and decline rapidly after that. In trisomic animals, these cells appear a day later than normal, and still represent a substantial proportion of the thymic population at 18 gd. In culture, the Ts16 thymuses also show a larger proportion of cells in this subset than do normal thymuses. This

TABLE 2
Expression of Thy-1, CD5, and CD44 During Embryonic Development and in Organ Culture

| Age[a] and genotype | Thy-1 | | | CD5 | | | CD44 | | |
|---|---|---|---|---|---|---|---|---|---|
| | $n$[b] | % positive ±S.D.[c] | $p$ | $n$[b] | % positive ±S.D.[c] | $p$ | $n$[b] | % positive ±S.D.[c] | $p$ |
| *In vivo* | | | | | | | | | |
| 13 Nor. | 9 | 53.0±20.3 | | 6 | 17.3±21.2 | | 4 | 85.8±39.3 | |
| Tri. | 5 | 22.4±19.8 | <0.05 | 4 | 13.7±13.7 | | 4 | 39.3±23.1 | <0.01 |
| 14 Nor. | 8 | 68.4±14.6 | | 4 | 20.1±6.3 | | 5 | 54.1±23.2 | |
| Tri. | 6 | 43.0±23.7 | <0.05 | 4 | 22.1±14.4 | | 5 | 67.8±9.5 | |
| 15 Nor. | 5 | 96.5±2.5 | | 5 | 21.5±3.6 | | 7 | 47.4±9.9 | |
| Tri. | 5 | 81.6±10.0 | <0.05 | 4 | 18.8±3.1 | | 5 | 55.0±10.4 | |
| 16 Nor. | 5 | 96.0±4.9 | | 5 | 53.9±14.4 | | 5 | 24.1±7.1 | |
| Tri. | 5 | 86.2±13.1 | [d] | 5 | 16.5±8.7 | <0.01 | 5 | 37.1±18.7 | |
| 17 Nor. | 5 | 93.5±7.9 | | 4 | 74.6±8.1 | | 4 | 25.4±14.3 | |
| Tri. | 5 | 84.4±11.3 | | 3 | 30.6±21.5 | <0.05 | 4 | 18.5±7.7 | |
| 18 Nor. | 7 | 99.3±0.5 | | 6 | 91.4±3.6 | | 4 | 8.3±8.6 | |
| Tri. | 4 | 89.2±8.1 | <0.01 | 6 | 48.2±27.5 | <0.01 | 4 | 4.8±2.0 | |
| *In vitro* | | | | | | | | | |
| 3 Nor. | 3 | 97.2±0.8 | | 4 | 57.6±2.9 | | 4 | 43.7±13.3 | |
| Tri. | 4 | 92.9±6.0 | | 4 | 22.6±6.6 | <0.01 | 4 | 40.5±3.2 | |
| 6 Nor. | 2 | 97.9±2.2 | | 4 | 81.2±3.0 | | 5 | 35.5±14.7 | |
| Tri. | 2 | 98.8±0.4 | | 4 | 62.1±2.9 | <0.01 | 5 | 11.8±4.9 | <0.01 |
| 9 Nor. | 2 | 97.5±0.9 | | 4 | 82.4±1.7 | | 5 | 43.5±10.9 | |
| Tri. | 2 | 97.2±1.3 | | 4 | 81.4±4.2 | | 4 | 18.6±10.8 | <0.05 |

[a]Age indicated for cultured thymuses is the number of days in culture.
[b]$n$=number of thymuses tested.
[c]S.D.=standard deviation.
[d]These values were significantly different ($p$<0.05) according to the Mann-Whitney $U$ test.

may indicate a partial block to thymocyte development that prevents or slows the progress of cells from the CD8+/CD4- subset to the CD8+/CD4+ subset. CD8-/CD4+ cells, which presumably represent the most mature cells in the thymus, are first evident after 6 days in culture and, in contrast to the other subsets, show little difference between normal and Ts16 thymuses. In contrast, there is no consistent difference between normal and Ts16 thymocytes in the proportion of cells expressing CD44, a marker for immature thymocytes (Table 2). In thymuses cultured *in vitro*, the percentage of cells expressing CD44 declines more rapidly in trisomic than in normal thymuses.

### Expression of T-cell Receptors

A smaller proportion of cells expressed CD3 in Ts16 as compared to normal thymuses (Table 4), again with some catching up *in vitro*. CD3+ cells in the thymus fall into two groups—CD8-/CD4- cells, which appear early in thymus development, and CD8/CD4 single-positive cells, which arise later. The results with CD44 indicated that early stages of thymocyte differentiation were not affected by the trisomy. Therefore, we were interested to know whether Ts16 affects both CD3-expressing subsets or only the later-appearing one. To examine this question, thymocytes were triple-labeled with CD8, CD4, and CD3. The proportion of cells expressing CD3 in the various CD8/CD4 subsets was the same in normal as in Ts16 thymuses in virtually all cases. However, the Ts16 CD8-/CD4- cells do show a small but consistent decrease in the percentage labeling with CD3 (Table 4). This indicates that production of the CD8-/CD4-/CD3+ population, which includes cells expressing the $\gamma/\delta$ T-cell receptor, is affected by Ts16, although perhaps not as much as the populations expressing CD8 and CD4.

To test this more directly, thymocytes were labeled with monoclonal antibody 536, which detects the V$\gamma$3 T-cell receptor chain. As shown in Table 4, the percentage of cells labeled with 536 is greater than or equal to that seen in the normal thymus throughout development. There is no indication of a delay in the onset of expression of this antigen, although the decline in this popu-

TABLE 3
Percentage of Cells in CD8/CD4 Defined Subsets During Embryonic Development and in Organ Culture

| Age[a] and genotype | | n | Subset | | | | | | | |
|---|---|---|---|---|---|---|---|---|---|---|
| | | | CD8+/CD4+ | | CD8-/CD4- | | CD8+/CD4- | | CD8-/CD4+ | |
| | | | Mean % ±S.D. | $p^b$ | Mean % ±S.D. | $p^b$ | Mean % ±S.D. | $p^b$ | Mean % ±S.D. | $p^b$ |
| *In vivo* | | | | | | | | | | |
| 15 | Nor. | 5 | 0.9±0.5 | | 93.9±2.6 | | 2.9±2.2 | | 2.3±0.6 | |
| | Tri. | 5 | 2.5±1.4 | <0.05[c] | 90.9±6.3 | | 2.9±1.7 | | 3.9±4.2 | |
| 16 | Nor. | 5 | 28.7±9.9 | | 56.4±10.9 | | 11.5±2.9 | | 3.4±2.9 | |
| | Tri. | 6 | 3.7±3.4 | <0.1 | 89.9±5.4 | <0.01 | 3.5±2.1 | | 2.9±0.8 | |
| 17 | Nor. | 4 | 62.7±8.3 | | 28.0±7.5 | | 6.1±3.2 | | 3.3±0.7 | |
| | Tri. | 4 | 7.3±7.0 | <0.01 | 74.2±11.1 | <0.01 | 15.2±3.8 | <0.01 | 3.3±1.7 | |
| 18 | Nor. | 7 | 79.0±8.1 | | 12.8±6.4 | | 5.8±2.7 | | 2.5±1.0 | |
| | Tri. | 5 | 31.3±27.7 | <0.01 | 52.8±36.9 | <0.05 | 13.5±10.5 | <0.1 | 2.3±1.8 | |
| *In vitro* | | | | | | | | | | |
| 3 | Nor. | 5 | 42.8±7.6 | | 37.0±8.1 | | 17.7±2.8 | | 2.5±0.8 | |
| | Tri. | 5 | 3.8±1.5 | <0.01 | 73.0±4.8 | <0.01 | 21.4±4.0 | | 1.8±0.8 | |
| 6 | Nor. | 4 | 66.9±6.0 | | 11.9±3.8 | | 8.3±2.7 | | 13.0±5.1 | |
| | Tri. | 4 | 51.8±1.6 | <0.05 | 19.4±1.7 | <0.01 | 19.4±2.6 | | 9.4±2.7 | |
| 9 | Nor. | 5 | 52.5±13.4 | | 21.2±12.3 | | 14.4±6.3 | | 12.0±5.8 | |
| | Tri. | 5 | 46.6±17.7 | | 24.8±16.9 | | 18.8±4.7 | | 9.8±2.7 | |

[a]Age indicated for cultured thymuses is the number of days in culture, with thymuses for cultures taken from 14-gd embryos.
[b]$p$ values were derived from Student's $t$ test.
[c]These values were not significantly different ($p$>0.05) according to the Mann-Whitney $U$ test.

lation near the end of development is apparently slowed.

### Thymic Reconstitution Experiments

Having shown that Ts16 significantly affected the differentiation of thymocytes, we were interested in determining whether the defect lay primarily in the lymphocytes themselves or in the thymic stroma. The technique of thymic reconstitution (Jenkinson et al., 1982) was used to address this question. In order to distinguish donor from host lymphocytes, cells or stroma from C57Bl/Ka mice (referred to hereafter as Ka), which carry the Thy-1.1 allele, were combined with the cells or stroma from the Ts16 mice or their normal littermates. These latter express Thy-1.2 that can be distinguished from Thy-1.1 by labeling with specific antibodies. In all subsequent experiments, a sample of lymphocytes from each reconstituted thymus was labeled with biotinylated Thy-1.2 antibody (rat IgG) followed by phycoerythrin-conjugated avidin as well as with Thy-1.1 antibody (mouse IgM) followed by fluoresceinated antimouse IgM. Any thymus showing a significant number of host-type cells (>50%) was excluded from consideration. In order to determine whether Ts16 cells or stroma produced lymphocytes that differed from normal, the cells derived from reconstituted thymuses were labeled with antibodies to CD8 and CD4. These markers were chosen because they showed the greatest and most consistent differences between normal and Ts16 thymuses. When cells from Ts16 animals and their normal littermates were used to reconstitute thymic lobes, equal numbers of cells were used to eliminate possible effects on development due to differences in the number of cells obtained from normal and Ts16 tissues.

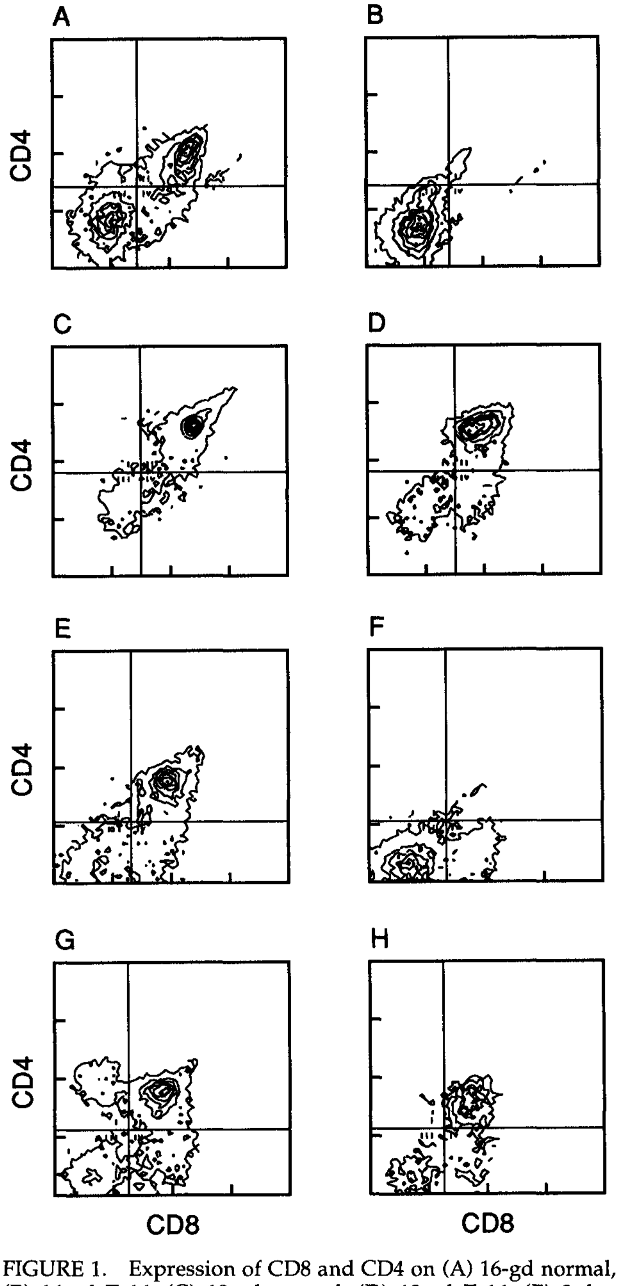

FIGURE 1. Expression of CD8 and CD4 on (A) 16-gd normal, (B) 16-gd Ts16, (C) 18-gd normal, (D) 18-gd Ts16, (E) 3-day cultured normal, (F) 3-day cultured Ts16, (G) 9-day cultured normal, and (H) 9-day cultured Ts16 thymocytes. Data are plotted on a log scale with a 4-decade range, with CD8 expression (FITC label) on the horizontal axis and CD4 expression (PE label) on the vertical axis.

Thymocytes from 14 gd fetal mice were used to reconstitute depleted thymic rudiments. The reconstituted thymuses were cultured for 6 days, then labeled with antibodies to CD8 and CD4, and analyzed by flow cytometry. Results are shown in Table 5 and Fig. 2. When Ka thymuses were reconstituted with thymocytes from Ts16 animals or their normal littermates, lymphocytes derived from trisomic cells show a significantly higher percentage of cells in the immature double-negative population and a lower percentage in the $CD8^+/CD4^-$ subset, indicating that the Ts16 thymocytes were delayed in their maturation even in a normal stromal environment.

Next, the reverse experiment was performed, in which Ka thymocytes were seeded into thymic lobes from Ts16 mice and their normal littermates. Again, some difference between normal and Ts16 thymic stroma was seen, but less than in the first experiment. Ts16, therefore, must affect the ability of the thymic stroma to support the development of thymocytes, but this defect may not be as great as that seen on the lymphocytes.

The numbers of cells derived from these reconstituted thymuses were also compared (Table 5). Although there was not a high level of significance because variability was very high, the Ts16 cells seeded into normal stroma did show some tendency to yield fewer cells after culture, whereas the normal cells in Ts16 stroma did not. This lends further support to the hypothesis that the lymphocytes are more affected than the thymic stroma by Ts16.

### DISCUSSION

These studies show defects in cell number and differentiation during the development of the trisomy 16 thymus. The trisomy causes a reduction in cell number throughout development and adversely affects the ability of the thymus to survive in organ culture. The appearance of mature thymocyte subsets is delayed, but the early stages of thymus development are less affected. Thymic reconstitution experiments indicate that Ts16 affects both the lymphocytes and the thymic stroma.

It had already been established (Epstein et al., 1985) that the Ts16 thymus is considerably smaller than that of the normal mouse throughout fetal development. There was some question as to whether this difference resulted from slower proliferation of thymocytes or fewer stem cells entering the thymus. The results presented here indicate that the latter explanation is the correct one, as the normal and trisomic thymus are already significantly different in cell number at 13 days and this difference increases only slightly during development, although there may be some decline of the Ts16 thymus at 18 gd when some of the trisomic fetuses appear to be dying. In culture, however, the Ts16 thymocytes do not grow as well as their normal counterparts, which may indicate that they are more sensitive to environmental changes or that they are more limited than normal cells in the length of time

TABLE 4
Percentage of Thymocytes Expressing CD3 and Vγ3 During Embryonic Development and in Organ Culture

| Age[a] and genotype | CD3 on total cells | | | CD3 on $CD8^-/CD4^-$ cells | | | Vγ3 on total cells | | |
|---|---|---|---|---|---|---|---|---|---|
| | *n* | % positive ±S.D. | *p*[b] | *n* | % positive ±S.D. | *p*[b] | *n* | % positive ±S.D. | *p*[b] |
| *In vivo* | | | | | | | | | |
| 14 Nor. | 3 | 0.7±0.4 | | | N.D. | | 6 | 0.6±0.5 | |
| Tri. | 3 | 2.5±3.8 | | | N.D. | | 6 | 1.7±1.1 | <0.05* |
| 15 Nor. | 5 | 7.0±2.1 | | | N.D. | | 2 | 2.2±0.3 | |
| Tri. | 6 | 1.9±1.3 | <0.01 | | N.D. | | 2 | 1.5±0.8 | |
| 16 Nor. | 5 | 6.8±4.3 | | 4 | 21.9±10.5 | | 3 | 1.6±0.4 | |
| Tri. | 5 | 3.1±0.6 | | 4 | 14.5±8.0 | | 3 | 1.1±0.9 | |
| 17 Nor. | 4 | 7.8±2.2 | | 4 | 9.0±2.2 | | 4 | 0.9±0.2 | |
| Tri. | 4 | 6.1±2.9 | | 4 | 8.5±3.0 | | 4 | 3.9±1.3 | <0.01 |
| 18 Nor. | 4 | 23.0±7.8 | | 5 | 13.5±1.2 | | 6 | 0.4±0.1 | |
| Tri. | 4 | 12.2±3.1 | <0.05[c] | 5 | 12.3±0.9 | | 5 | 2.0±0.8 | <0.01 |
| *In vitro* | | | | | | | | | |
| 3 Nor. | 4 | 23.3±8.0 | | 6 | 11.5±4.8 | | | | |
| Tri. | 4 | 13.1±2.9 | <0.1 | 7 | 24.2±10.6 | <0.01 | | | |
| 6 Nor. | 4 | 27.1±7.8 | | 4 | 38.3±7.4 | | | | |
| Tri. | 4 | 18.7±5.0 | | 4 | 26.5±12.1 | | | | |
| 9 Nor. | 4 | 34.8±7.4 | | 3 | 39.4±8.3 | | | | |
| Tri. | 4 | 30.9±9.9 | | 4 | 29.3±10.5 | | | | |

[a]Age indicated for cultured thymuses is the number of days in culture, with thymuses for cultures taken from 14-gd embryos.
[b]*p* values were derived from Student's *t* test.
[c]These values were not significantly different ($p$>0.05) according to the Mann-Whitney *U* test.

TABLE 5
Cell Number and CD8/CD4 Expression of Reconstituted Thymuses[a]

| Host thymus | Ka | Ka | | Rb[b] normal | Rb[b] Ts16 | |
|---|---|---|---|---|---|---|
| Source of lymphocytes | Rb[b] normal | Rb[b] Ts16 | *p*[c] | Ka | Ka | *p*[c] |
| *n* | 3 | 4 | | 3[d] | 3 | |
| Cell number ±S.D. | $1.4\times10^4$ $\pm8.0\times10^3$ | $4.5\times10^3$ $\pm2.1\times10^3$ | <0.1 | $3.5\times10^4$ $\pm2.1\times10^4$ | $5.4\times10^4$ $\pm5.0\times10^4$ | |
| | Mean% ±S.D. | Mean% ±S.D. | | Mean% ±S.D. | Mean% ±S.D. | |
| $CD8^+/CD4^+$ | 10.4±2.7 | 9.8±1.2 | | 9.1±1.9 | 7.3±2.4 | |
| $CD8^-/CD4^-$ | 52.5±3.3 | 71.6±3.8 | <0.01 | 52.7±3.6 | 63.1±4.3 | <0.05 |
| $CD8^+/CD4^-$ | 32.5±6.9 | 14.7±3.8 | <0.01 | 34.3±3.7 | 25.9±9.0 | |
| $CD8^-/CD4^+$ | 4.6±1.0 | 4.0±2.4 | | 3.9±1.5 | 3.7±2.3 | |

[a]Thymocytes were taken from 14-gd embryos and reconstituted thymuses were cultured for 6 days.
[b]Rb indicates animals derived from cross using Rb(6.16/16.17) male.
[c]*p* values were derived from Student's *t* test.
[d]Cell numbers were determined for only two of these samples.

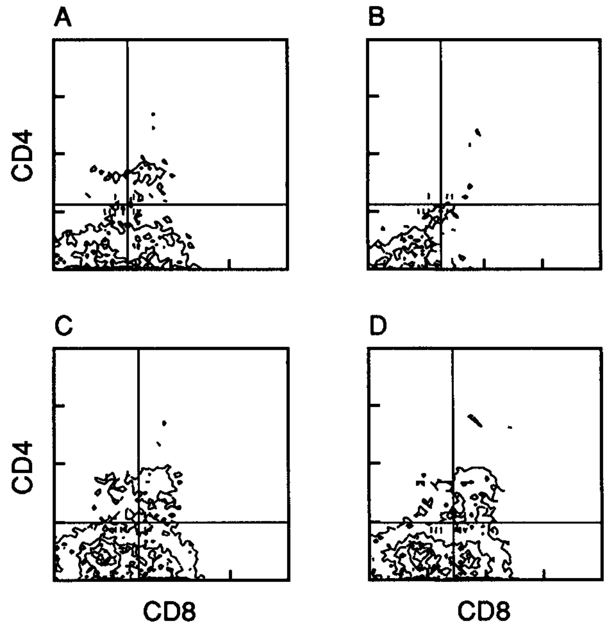

FIGURE 2. Expression of CD8 and CD4 on cells from depleted thymuses reconstituted with 14-day thymocytes and cultured for 6 days. (A) Ka thymuses seeded with normal thymocytes. (B) Ka thymuses seeded with Ts16 lymphocytes. (C) Normal thymuses seeded with Ka lymphocytes. (D) Ts16 thymuses seeded with Ka lymphocytes. Data are plotted on a log scale with a 4-decade range, with CD8 expression (FITC label) on the horizontal axis and CD4 expression (PE label) on the vertical axis.

they can survive or the number of divisions they can undergo.

When Ts16 thymocytes were labeled with antibodies to markers characteristic of mature T cells, the results generally indicated developmental retardation. The patterns of staining with CD5, CD8, and CD4 were particularly clear in this regard (Tables 2 and 3; Fig. 1). Expression of each of these markers showed a delay of 1–2 days throughout fetal development, and cultured thymuses were able to "catch up" to their normal counterparts. This pattern of retardation also tends to be true for CD3 expression (Table 4).

Not all differentiation markers show such a clear-cut pattern of developmental retardation. Thy-1 is expressed on virtually 100% of thymocytes through most of fetal development in normal mice. In trisomic animals, expression of this marker appears to reach a constant level of expression at the same time as in normal thymuses. However, in the Ts16 mice, a small population of nonstaining cells remains throughout development. Interestingly, the results seen for Thy-1 *in vitro* are quite different, as the Ts16 thymocytes show the same high levels of expression as in the normal thymuses. This would seem to indicate either that a factor present in the trisomic animal is preventing the maturation of cells in the thymus, or a subset of cells that do not express differentiation markers migrates into the trisomic thymus *in vivo* after the stage at which the thymus is removed for culture in these experiments. The second possibility seems likely to be true because these cultures were taken from the animals before the second wave of precursors is believed to migrate into the thymus (Owen and Jenkinson, 1981).

These results largely corroborate those of Berger and Epstein (1989). They examined expression of Thy-1, CD4, CD8, CD5, and CD3 during fetal development with largely similar results. The results seen in thymic organ culture, however, were somewhat different, which may be because thymuses were taken for culture beginning at 15 or 17 gd rather than 14 gd. Data were obtained from thymuses cultured for as long as 17 days (beginning at 15 gd), which may indicate that later stem-cell immigrants have entered the Ts16 thymus at this point and are more able to survive in culture than the first-wave cells present in the 14-day thymus. Alternatively, it may be that the smaller size and reduced cellularity of the Ts16 thymus are the important factors in its lessened ability to survive *in vitro* and that by allowing one more day of growth *in vivo*, it is possible to circumvent this problem. Expression of CD3, CD4, and CD8 did catch up to normal in-cultured thymuses in both studies.

Antigens expressed only on immature or early appearing thymocyte subsets, such as CD44 and V$\gamma$3, are apparently less affected by Ts16. The fact that the loss of CD44 expression seems completely unaffected (Table 2) supports the idea that this is a very early event in thymocyte differentiation, as it seems unlikely that cells already delayed in their development (by criteria such as the expression of, for example, Thy-1) would then undergo a later developmental event with normal timing. Because the early expression of both V$\gamma$3 and CD3 also appear to be unaffected, the early $\gamma/\delta$ cells (and, possibly, the dendritic epidermal cells derived from them; Havran and Allison, 1990) may be completely normal in Ts16 mice.

There are two possible explanations for the relative lack of effect on more immature thymocyte subsets. One is that development is able to proceed normally up to a certain point, but is delayed after this, possibly due to some specific regulatory mechanism altered by the trisomy. The other is that, if development is really taking place at a slower rate (as opposed to occurring with a delay compared to normal), it would be expected that there would be a greater disparity between the timing of later events than that of earlier ones. Although it is difficult to distinguish between these possibilities, the lack of effect on CD44 expression would indicate that there is an early stage unaffected by Ts16. The fact that there is no more difference between the normal and trisomic thymuses in expression of this marker at 18 gd than there is at 14 gd makes it more likely that the trisomy affects a particular point in thymocyte development.

In addition to the defect seen in late stages of maturation of the Ts16 thymus there is evidence for effects of the trisomy on stem-cell proliferation and/or survival. Because the Ts16 thymus contains fewer cells from the beginning of its development, there must be a defect that affects the proliferation or formation of hematopoietic stem cells before they enter the thymus. The decline in cell number after long-term culture of the Ts16 thymus, as well as the decrease in the number of CD44-expressing cells seen in cultured thymic lobes, may indicate that there is also a problem in the ability of thymic stem cells to survive in organ culture.

The thymic reconstitution experiments were performed to address the question of stem-cell competence more directly, but the results obtained were somewhat ambiguous. Ts16 lymphocytes did seem to be impaired compared to normal in their ability to differentiate in a normal thymic stroma. There was also some indication of an effect of Ts16 on the stroma that may be impaired in its ability to support thymocyte differentiation. It is possible that the relatively small differences seen in reconstituted thymuses as compared to untreated cultured thymuses were because the reconstitution was performed with equal numbers of cells for normal and Ts16 tissues. In normal thymic development, lymphokines produced by the thymocytes themselves may be important for T-cell development (Jenkinson et al., 1987; Skinner et al., 1987; Carding et al., 1989), so it is possible that the problems seen in the Ts16 thymus are due partially to an insufficient number of cells to produce the required concentration of these factors.

It is difficult to relate these results directly to the immune defects seen in Down Syndrome, as few studies of DS have looked at fetal tissue. The expression of some differentiation markers on the DS fetal thymus has been examined (Cossarizza et al., 1989). No differences between normal and DS thymuses were seen, but only two cases of each type were examined and the variability between them was very high. Another study, which looked at thymocytes from a larger number of young children with DS (Larocca et al., 1988) did find significant decreases in the expression of several thymocyte antigens, including CD3, CD4, and CD8.

Of course, it would be most important if the Ts16 phenotype proved to have any relevance to the clinical problems of the DS immune system. If immature thymocytes (i.e., those not expressing differentiation antigens) persist in DS as they do in Ts16, they may be involved in the development of leukemia. These thymocytes could not account for all of the proliferative abnormalities seen in DS, as many different types of lymphocytic and nonlymphocytic leukemias are seen (Fong and Brodeur, 1987). It would be of interest to determine whether other types of hematopoietic cells show similar developmental delays in Ts16 and/or DS, which could indicate that various types of immature hematopoietic cells exist and have the potential to become leukemoid in DS. The observed defects in T-cell functions seen in DS probably could not be studied in cells derived directly from Ts16 mice, because mature CD4 and CD8 single-postive cells are not produced by the time these animals die. However, these cells are produced in organ cultures, which could be used as a source of material to test the functional capacities of Ts16 T cells. In addition, such cultures could be used for testing the effects of lymphokines or other factors on the differentiation of Ts16 cells.

The usefulness of any of these approaches to relating the phenotype of the Ts16 mouse to the clinical manifestations of DS is limited because the mice do not survive past birth. There are several approaches that may be useful in circumventing this problem. One already mentioned is that of making chimeric mice by fusing Ts16 and normal blastocysts. Studies using this method (Cox et al., 1984; Gearhart et al., 1986) have indicated that Ts16 hematopoietic cells are at a competitive disadvantage compared to normal cells in these chimeras, indicated by a deficiency of Ts16 cells in the thymus and a postnatal decrease in Ts16 cells in the blood. This fact, of course, makes it difficult to use such animals to study the functions of Ts16 immune-system cells, although the use of appropriate genetic markers (such as immunologically distinct alleles of thymocyte antigens) may make it possible to distinguish cells from the Ts16 blastocyst fusion partner by flow cytometry.

An alternative to the use of chimeras is to genetically alter mice so that they carry only parts of the chromosome in extra copies. Such animals may be expected to have fewer defects than those carrying the entire extra chromosome and therefore to survive longer. In addition, this approach would avoid the problem of how murine genes present on chromosome 16 but not shared with human chromosome 21 might affect development of Ts16 mice or cells derived from them. The human genomic sequence of SOD-1, one of the genes present on both mouse chromosome 16 and human chromosome 21, has been injected into mouse eggs to produce transgenic mice (Epstein et al., 1987). These mice express increased levels of the enzyme, but appear grossly normal. However, they do display alterations in neuromuscular junctions of the tongue similar to those seen in DS (Avraham et al., 1988). These results suggest that it may be possible to mimic specific phenotypes of DS in mice transgenic for genes found on human chromosome 21. However, they also suggest that the presence of one such extra gene is not enough to produce a broad spectrum of the phenotypic alterations associated with DS. A more accurate model would be a mouse carrying an extra copy of the entire chromosome segment responsible for the DS phenotype. It may be possible to create such animals by the injection of microdissected human chromosome segments into fertilized eggs to produce "transomic" mice (Richa and Lo, 1989). If viable mice carrying this portion of the human chromosome could be produced and were viable, they would be ideal candidates for a viable DS model in mice.

### MATERIALS AND METHODS

### Mice

Strains Rb(6.16)24Lub and Rb(16.17)8Lub, carrying Robertsonian translocation chromosomes, were obtained from Jackson Laboratories (Bar Harbor, Maine) and maintained as breeding stocks in our laboratory. Females of strain C57B1/6 were obtained from Jackson or bred in our laboratory. Breeding males were produced by crossing the two Robertsonian strains. These males were caged with C57B1/6 females and timed pregnancies were obtained by checking for vaginal plugs (plug day=0).

### Reagents

Monoclonal antibodies to Thy-1.2 (fluoresceinated or biotinylated), CD5 (fluoresceinated), CD8 (fluoresceinated or biotinylated), and CD4 (phycoerythrin conjugated), as well as allophycocyanin (APC)-conjugated avidin were obtained from Becton-Dickinson (Mountain View, California). Phycoerythrin-conjugated avidin was obtained from Vector (Burlingame, California). Monoclonal antibodies to CD44 (1M7.8.1; Trowbridge et al., 1982), from Robert Hyman, Salk Institute, (San Diego, California); CD3 (145-2C11; Leo et al., 1987), from Jeffrey A. Bluestone, National Cancer Institute, (Bethesda, Maryland); and Thy-1.1 (TIB 100, American Type Culture Collection) were produced in our laboratory as culture supernatants from James P. Allison. Fluoresceinated secondary reagents used were goat antirat IgG and goat antimouse IgM (TAGO, Inc., Burlingame, California) and goat antihamster IgG (Cappel, Organon Teknika Corp., West Chester, Pennsylvania).

### Generating Trisomic Animals

Pregnant females were sacrificed and fetuses tentatively scored as normal or trisomic based on edema and condition of eyelids (trisomics develop severe edema and their eyes usually do not close). Tissues to be used were dissected from all putative trisomics and from an equal number of normal littermates to use as controls.

### Karyotyping

Liver cells (13 gd or older animals) or yolk sac cells (12 gd) were used for karyotyping to verify the visual diagnosis. Karyotypes were attempted for all animals used, but occasionally samples were lost or mitotic cells were not found in the samples (This latter problem occurred most commonly in fetuses from later gestational stages, when Ts16 animals are very easy to distinguish by phenotype.) Nearly all (>90%) of animals were karyotyped, and the results always confirmed the diagnoses based on phenotype. Karyotyping was performed according to standard methods (Hogan et al., 1986). Cells were incubated for 2–12 hr (longer times were necessary for tissues from older fetuses) in RPMI+10% FBS+0.3 $\mu$g/ml colcemid+antibiotics+5 $\mu$g/ml LPS. They were then treated with 0.075 M KCl, fixed in 3:1 methanol:acetic acid, and stored at 4 °C. Suspensions were then dropped onto a slide, air dried, and stained with Giemsa (diluted 1:50). Slides were viewed under immersion oil on a Zeiss microscope at 1250× magnification.

### Flow Cytometric Analysis

Thymuses were manually dissociated with fine forceps, filtered through a 15-$\mu$ Nitex filter, counted in a hemocytometer, and resuspended in FACS buffer (PBS+1% BSA+0.1% sodium azide). Cells were incubated with antibody, lectin, or avidin for 30 min. When more than one labeling step was required, cells were washed with an excess of FACS buffer between incubations.

The cells were analyzed on a Becton-Dickinson FACStar Plus flow cytometer. Fluorescein and phycoerythrin were excited with a 488-nm argon laser line and APC with a 647-nm krypton laser line. Dead and nonlymphocytic cells were excluded on the basis of forward light scatter.

### Thymus Culture

Thymus culture was performed according to a method derived from that of Jenkinson et al. (1987) and modified in this laboratory. Culture dishes were prepared by placing 0.8-$\mu$ Nuclepore filters (Nuclepore, Pleasanton, California) on slices of Gelfoam gelatin sponges (Upjohn, Kalamazoo, Michigan) (approx. 1 mm thickness) in 35-mm tissue-culture dishes. (Both sponges and filters were preboiled twice.) 1 ml of culture medium (RPMI+10% FBS+antibiotics+0.01 M HEPES+0.0035% $\beta$-mercaptoethanol) was added to each dish. Thymic lobes were dissected sterilely from fetuses of 14 gestational days (gd), placed on the filters, and incubated at 37 °C with 5% $CO_2$ for the desired length of time.

To reconstitute thymuses, cultures were set up as before except that the culture medium contained 1.35 mM deoxyguanosine (dGuo), which kills lymphocytes while leaving the thymic stroma intact (Jenkinson et al., 1982). After 3 days in culture, these thymuses were washed extensively in medium without dGuo. Thymocytes were prepared by manual dissection as described for flow cytometry. These cells were counted with a hemocytometer and adjusted to the desired cell concentration. Cell suspensions obtained by these methods were placed in wells of a Terasaki plate (30 $\mu$l per well). One depleted thymic lobe was added to each well, and these were cultured as hanging drops for 24 hr. After this time, the thymuses were transferred to filters and cultured as described before.

### Data Analysis and Statistics

Flow cytometric data analysis was performed using the Becton-Dickinson Disp4 and Disp2d programs. In order to determine the percent positive of single-labeled samples versus controls, gates were set based on control histograms and applied to the labeled samples. The corrected percent positive was calculated according to the following formula:

$$\% \text{ labeled} = 100 \times \frac{\% \text{ positive (experimental)} - \% \text{ positive (control)}}{100 - \% \text{ positive (control)}}$$

Data in the tables are given as the mean plus or minus the standard deviation, with $p$ values (for the difference between normal and trisomic thymuses of the same age and experimental treatment) determined by Student's $t$-test. Due to the inherent variability of results, the data were also examined using the Mann-Whitney $U$ test as a check on the validity of the statistical analysis. In nearly all cases (exceptions are marked in the tables), differences determined to be significant at the 0.05 level by the $t$ test were also significant by the Mann-Whitney $U$ test. $p$ values are only shown for cases in which $p<0.1$.

### ACKNOWLEDGMENTS

The authors would like to thank L. Morrissey, J. Bielich, and L. Kubai for excellent technical assistance and C.-P. Liu and A. Globerson for helpful discussions. We also appreciate the reagents generously provided by Dr. J. A. Bluestone (the anti-CD3$\varepsilon$ mAb 145-2C11) and Dr. J. P. Allison (the anti-TCR V$\gamma$3 mAb 536).

This work was supported in part by funds from NSF grant DCB 86-2751. J.L.E. was supported by an NIH predoctoral training grant stipend (T32HD7118).

*(Received November 11, 1991)*

*(Accepted November 18, 1991)*

### REFERENCES

Antonarakis S.E., Warren A.C., McCormick M. K., Lewis J.G., Hieter P.A., and Chakravarti, A. (1989). Molecular mapping of chromosome 21 and the region responsible for Down Syndrome. *In:* Molecular and cytogenetic studies of non-disjunction, Hassold T.J., and Epstein C.J. Eds. (New York: Alan R. Liss, Inc.), pp. 29–43.

Avraham K.B., Schickler M., Sapoznikov D., Yarom R., and Groner Y. (1988). Down's Syndrome: Abnormal neuromuscular junction in tongue of transgenic mice with elevated levels of human Cu/Zn-superoxide dismutase. Cell **54:** 823–829.

Berger C.N., and Epstein C.J. (1989). Delayed thymocyte maturation in the trisomy 16 mouse fetus. J. Immunol. **143:** 389–396.

Carding S.R., Jenkinson E.J., Kingston R., Hayday A.C., Bottomly K. and Owen J.J.T. (1989). Developmental control of lymphokine gene expression in fetal thymocytes during T-cell ontogeny. Proc. Natl. Acad. Sci. USA **86:** 3342–3345.

Cossarizza A., Monti D., Montagnani G., Forabosco A., Dagna-Bricarelli F., and Franceschi C. (1989). Fetal thymic differentiation in Down's syndrome. Thymus **14:** 163–170.

Cox D.R., Smith S.A., Epstein L.B., and Epstein C.J. (1984). Mouse trisomy 16 as an animal model of human trisomy 21 (Down Syndrome): Production of viable trisomy 16–diploid mouse chimeras. Dev. Biol. **101:** 416–424.

Crispe I.N., and Bevan M.J. (1987). Expression and functional significance of the J11d marker on mouse thymocytes. J. Immunol. **138:** 2013–2018.

Epstein C.J. (1988). Mouse model systems for the study of aneuploidy. *In:* Aneuploidy, part B: Induction and test systems, Vig B.K., and Sandberg A.A. Eds. (New York: Alan R. Liss, Inc.), pp. 257–271.

Epstein C.J., Avraham K.B., Lovett M., Smith S., Elroy-Stein O., Rotman G., Bry C., and Groner Y. (1987). Transgenic mice with increased Cu/Zn-superoxide dismutase activity: Animal model of dosage effects in Down syndrome. Proc. Natl. Acad. Sci. USA **84:** 8044–8048.

Epstein C.J., Cox D.R., Epstein L.B., and Magnuson T.R. (1984). Animal models for human chromosome disorders. *In:* Research perspectives in cytogenetics, Sparkes R.S., and de la Cruz F.F. Eds. (Baltimore: University Park Press), pp. 75–95.

Epstein C.J., Hofmeister B.G., Yee D., Smith S.A., Philip R., Cox D.R., and Epstein L.B. (1985). Stem cell deficiencies and thymic abnormalities in fetal mouse trisomy 16. J. Exp. Med. **162:** 695–712.

Fong C.-T., and Brodeur G.M. (1987). Down's Syndrome and leukemia: Epidemiology, genetics, cytogenetics and mechanisms of leukemogenesis. Cancer Genet. Cytogenet. **28:** 55–76.

Fryers T. (1986). Survival in Down's Syndrome. J. Ment. Defic. Res. **30:** 101–110.

Gearhart J.D., Singer H.S., Moran T.H., Tiemeyer M., Oster-Granite M.L., and Coyle J.T. (1986). Mouse chimeras composed of trisomy 16 and normal (2N) cells: Preliminary studies. Brain Res. Bull. **16:** 815–824.

Gropp A. (1982). Value of an animal model for trisomy. Virchows Arch. [Pathol. Anat.] **395:** 117–131.

Gropp A., Kolbus U., and Giers D. (1975). Systematic approach to the study of trisomy in the mouse. II. Cytogenet. Cell Genet. **14:** 42–62.

Havran W.L., and Allison J.P. (1990). Origin of Thy-1+ dendritic epidermal cells of adult mice from fetal thymic precursors. Nature **344:** 68–70.

Herbst E.W., Gropp A., Neilsen K., Hoppe H., Freyman M., and Pluznik D.H. (1982). Reduced ability of mouse trisomy-16 stem cells to restore hematopoiesis in lethally irradiated animals. *In:* Experimental hematology today–1982, Baum S.J., Ledney G.D., and Theirfelder, S. Eds. (Basel: Karger), pp. 119–126.

Hogan B., Constantini F., and Lacy E. (1986). Manipulating the mouse embryo: a laboratory manual (Cold Spring Harbor, NY: Cold Spring Harbor Laboratory).

Jenkinson E.J., Franchi L.L., Kingston R., and Owen J.J.T. (1982). Effect of deoxyguanosine on lymphopoiesis in the developing thymus rudiment *in vitro*: Application in the production of chimeric thymus rudiments. Eur. J. Immunol. **12:** 262–271.

Jenkinson E.J., Kingston R., and Owen J.J.T. (1987). Importance of Il-2 receptors in intra-thymic generation of cells expressing T-cell receptors. Nature **329:** 160–162.

Larocca L.M., Piantelli M., Valitutti S., Castellino F., Maggiano N., and Musiani P. (1988). Alterations in thymocyte subpopulations in Down's syndrome (Trisomy 21). Clin. Immunol. Immunopathol. **49:** 175–186.

Leo O., Foo M., Sachs D.H., Samelson L.E., and Bluestone J.A. (1987). Identification of a monoclonal antibody specific for a murine T3 polypeptide. Proc. Natl. Acad. Sci. USA **84:** 1374–1378.

Miyabara S., Gropp A., and Winking H. (1982). Trisomy 16 in the mouse fetus associated with generalized edema and cardiovascular and urinary tract anomalies. Teratology **25:** 369–380.

Montagna D., Maccario R., Ugazio A.G., Nespoli L., Pedroni E., Faggiano P., and Burgio G.R. (1988). Cell-mediated cytotoxicity in Down syndrome: Impairment of allogeneic mixed lymphocyte reaction, NK and NK-like activities. Eur. J. Pediatr. **148:** 53–57.

Nair M.P.N., and Schwartz S.A. (1984). Association of decreased T-cell-mediated natural cytotoxicity and interferon production in Down's syndrome. Clin. Immunol. Immunopathol. **33:** 412–424.

Noble R.L., and Warren R.P. (1988). Analysis of blood cell populations, plasma zinc and natural killer cell activity in young children with Down's syndrome. J. Ment. Defic. Res. **32:** 193–201.

Owen J.J.T. and Jenkinson E.J. (1981). Embryology of the lymphoid system. Prog. Allergy **29:** 1–34.

Rahmani Z., Blouin J.-L., Creau-Goldberg N., Watkins P.C., Mattei J.-F., Poissonier M., Prieur M., Chettouh Z., Nicole A., Aurias A., Sinet P.-M., and Delabar J.-M. (1989). Critical role of the *D21S55* region on chromosome 21 in the pathogenesis of Down syndrome. Proc. Natl. Acad. Sci. USA **86:** 5958–5962.

Raziuddin S., and Elawad M.E. (1990). Immunoregulatory CD4+CD45R+ suppressor/inducer T lymphocyte subsets and impaired cell-mediated immunity in patients with Down's syndrome. Clin. Exp. Immunol. **79:** 67–71.

Reeves R.H., Gearhart J.D., and Littlefield J.W. (1986). Genetic basis for a mouse model of Down Syndrome. Brain Res. Bull. **16:** 803–814.

Richa J., and Lo C. (1989). Introduction of human DNA into mouse eggs by injection of dissected chromosome fragments. Science **245:** 175–177.

Sassaman E.A. (1982). Biomedical aspects in Down Syndrome: Immunology. *In:* Down Syndrome: Advances in biomedicine and the behavioral sciences, Pueschel S.M., and Rynders J.E. Eds. (Cambridge, MA: Ware Press), pp. 229–233.

Sinet P.-M., Couturier J., Dutrillaux B., Poissonier M., Raoul O., Rethore M.-O., Allard D., Lejeune J., and Jerome H. (1976). Trisomie 21 et superoxyde dismutase-I (IPO-A): Tentative de localisation sur la sous bande 21q22.1. Exp. Cell Res. **97:** 47–55.

Skinner M., Le Gros G., Marbrook J., and Watson J.D. (1987). Development of fetal thymocytes in organ cultures: Effect of interleukin 2. J. Exp. Med. **165:** 1481–1493.

Springer T., Galfre G., Secher D.S., and Milstein C. (1978). Monoclonal xenogeneic antibodies to murine cell surface antigens: Identification of novel leukocyte differentiation antigens. Eur. J. Immunol. **8:** 539–551.

Summitt R.L. (1981). Chromosome 21: Specific segments that cause the phenotype of Down syndrome. *In:* Trisomy 21 (Down Syndrome), de la Cruz F.F., and Gerald P.S. Eds. (Baltimore: University Park Press), pp. 225–235.

Trowbridge I.S., Lesley J., Schulte R., Hyman R., and Trotter J. (1982). Biochemical characterization and cellular distribution of a polymorphic, murine cell-surface glycoprotein expressed on lymphoid tissues. Immunogenet. **15:** 299–312.

